# Erosion of the healthy soldier effect in veterans of US military service in Iraq and Afghanistan

**DOI:** 10.1186/s12963-015-0040-6

**Published:** 2015-03-18

**Authors:** Mary J Bollinger, Susanne Schmidt, Jacqueline A Pugh, Helen M Parsons, Laurel A Copeland, Mary Jo Pugh

**Affiliations:** South Texas Veterans Health Care System, 7400 Merton Minter Blvd, San Antonio, Texas USA; Department of Medicine, Division of Hospital Medicine, University of Texas Health Science Center, San Antonio, Texas USA; Department of Epidemiology and Biostatistics, University of Texas Health Science Center, San Antonio, Texas USA; Central Texas Veterans Health Care System, Department of Veterans Affairs, 1901 Veterans Memorial Drive, Temple, Texas 76504 USA; Center for Applied Health Research, Baylor Scott & White Health, 2102 Birdcreek Drive, Temple, Texas 76502 USA

**Keywords:** Veterans/statistics & numerical data, Mortality, Healthy soldier effect

## Abstract

**Background:**

This research explores the healthy soldier effect (HSE) – a lower mortality risk among veterans relative to the general population—in United States (US) veterans deployed in support of operations in Iraq and Afghanistan (OEF/OIF/OND). While a HSE has been affirmed in other OEF/OIF/OND populations, US veterans of OEF/OIF/OND have not been systematically studied.

**Methods:**

Using US Department of Veterans Affairs (VA) administrative data, we identified veterans who (1) had been deployed in support of OEF/OIF/OND between 2002 and 2011 and (2) were enrolled in the VA health care system. We divided the VA population into VA health care utilizers and non-utilizers. We obtained Department of Defense (DOD) administrative data on the OEF/OIF/OND population and obtained VA and DOD mortality data excluding combat deaths from the analyses. Indirect standardization was used to compare VA and DOD cohorts to the US population using total population at risk to compute the Standardized Mortality Ratio (SMR). A directly standardized relative risk (DSRR) was calculated to enable comparisons between cohorts. To compare VA enrollee mortality on military specific characteristics, we used a DOD population standard.

**Results:**

The overall VA SMR of 2.8 (95% Confidence Interval [CI] 2.8-2.9), VA utilizer SMR of 3.2 (95% CI 3.1-3.3), VA non-utilizer SMR of 0.9 (95% CI 0.8-1.1), and DOD SMR of 1.5 (95% CI 1.4-1.5) provide no evidence of a HSE in any cohort relative to the US standard population. Relative to DOD, both the total VA population SMR of 2.1 (95% CI 2.0-2.2) and the SMR for VA utilizers of 2.3 (95% CI 2.3-2.4) indicate mortality twice what would be expected given DOD mortality rates. In contrast, the VA enrollees who had not used clinical services had 40% lower than expected mortality relative to DOD.

**Conclusions:**

No support was found for the HSE among US veterans of OEF/OIF/OND. These findings may be attributable to a number of factors including post-deployment risk-taking behavior, an abbreviated follow up period, and the nature of the OEF/OIF/OND conflict.

## Background

Between 2002 and 2011, more than 4.6 million US service members were deployed to support Operation Enduring Freedom (OEF), Operation Iraqi Freedom (OIF), and Operation New Dawn (OND) (OEF/OIF/OND) activities [1]. With increasing attention focused on military and veteran suicide, post-traumatic stress disorder, and other mental health conditions, as well as recovery from and readjustment to severe combat injuries, we sought to discover whether OEF/OIF/OND Veterans were less healthy and at higher risk for death than their counterparts in the general US population. Previous research indicates veterans are generally healthier than their civilian counterparts; however, this cohort of veterans seemed to be facing unique problems that might lead to decreased survivability relative to the general population.

Since World War II, researchers have looked to explain the lower mortality observed in veterans focusing on the selection effects of entry standards into the armed forces. Recruits are generally young and fit with very low rates of chronic disease (e.g., healthy soldier effect, HSE) [2,3]. More recently, a healthy warrior effect has been identified among deployed military members with researchers noting that good health is a prerequisite for deployment (e.g., healthy warrior effect).

Research examining the Healthy Worker Effect (HWE) upon which the HSE is based has found that the effect is modified by age, sex, length of employment, race, and occupation. The effect is strongest at youngest ages, but increasing employment duration increases the effect. In addition, the effect appears to be strongest for women, [4] greater for non-whites, [5] and increased for physically demanding jobs [6].

Quantifying the HSE, Seltzer and Jablon [2] found mortality in a World War II cohort to be 13% to 30% lower than the general US population but also found that the mortality gap decreased over time. Kang and colleagues [3] found that the mortality of Gulf War veterans compared to military members serving during the same time who were not deployed to the Persian Gulf was slightly but significantly higher. Relative to the US population, however, both groups had significantly lower mortality, more than half of what was predicted after adjustment for age, sex, race, and year of death. The expected mortality advantage of women compared to men was not consistently observed either in the comparisons between the two military cohorts or between the cohorts and the US population. This study and subsequent studies [7] found that post-deployment mortality was lower for all-cause deaths but higher for deaths due to external causes, primarily accidents, although the higher mortality declined over time [8].

The HSE has been affirmed in military cohorts from Australia, [9] Norway, [10] and New Zealand [11]. In the Australian Korean War cohort study, the HSE was found to persist up to 30 years following service for all-cause mortality, although the HSE varied by cause of death with deaths from external causes elevated for up to 30 years. In Australian Vietnam veterans, the HSE for all-cause mortality lasted more than 30 years, and the excess of deaths for external causes persisted only up to 10 years. Current studies on the HSE focused on disability [12,13] and psychological health [14,15] have also found evidence for a HSE. A 2013 Australian study of OEF/OIF/OND veterans and a French study of military males serving between 2006–2010 both found all-cause mortality still lower compared to their respective general populations [16,17].

Thus, the research to date supports a HSE in all-cause mortality; however, the focus has been primarily on veterans from previous eras or veterans from other countries. No assessment of the HSE has been done in US OEF/OIF/OND veterans, however. Two characteristics distinguish these veterans from veterans of previous wars. First, after 12 years, some soldiers are still deployed in combat theaters. Second, medical and technological advances improved survival from injuries that would have been fatal in previous conflicts, meaning that many more veterans will be living with some type of disability compared to previous war cohorts. In contrast with OEF/OIF/OND veterans from other countries and also different from previous conflicts, US military personnel strength was inadequate to meet conflict demands. To compensate, the US required more frequent deployments for longer durations with a heavier reliance on Guard and Reserve forces who were ill-prepared for such experiences. These unique characteristics suggest that the mortality experiences of the US OEF/OIF/OND cohort may be very different from previous cohorts.

We therefore undertook this study to explore whether the mortality experience of OEF/OIF/OND veterans differed from that of previous veteran cohorts by examining the ways in which the HSE operates in Veterans Administration (VA) enrollees and Department of Defense (DOD) active duty service members compared to the US population. Building upon prior work, we assess mortality differences between the general US population, 3 VA cohorts (enrolled in VA health care, with utilization, and without utilization), and an active-duty military cohort (active duty military/activated Guard/Reserve).

## Materials and methods

### Data

VA data were extracted from the VA OEF/OIF/OND Roster file of veterans deployed in support of Afghanistan and Iraq combat operations since October 2001 and who have (1) been discharged from active duty, (2) have an existing relationship with the VA, and (3) have been involved in the OEF/OIF/OND mission either within or outside of a designated combat zone [18]. These data were merged with VA Mini Vital Status mortality data. We also obtained data from DOD on personnel who had served in support of OEF/OIF/OND at any point during 2002–2011 from the Defense Manpower Data Center (DMDC) Reporting System (DRS). DRS data include all active-duty personnel, as well as activated National Guard and Reserve forces, all of whom may still be serving on active duty.

### Study population

**Figure 1:**
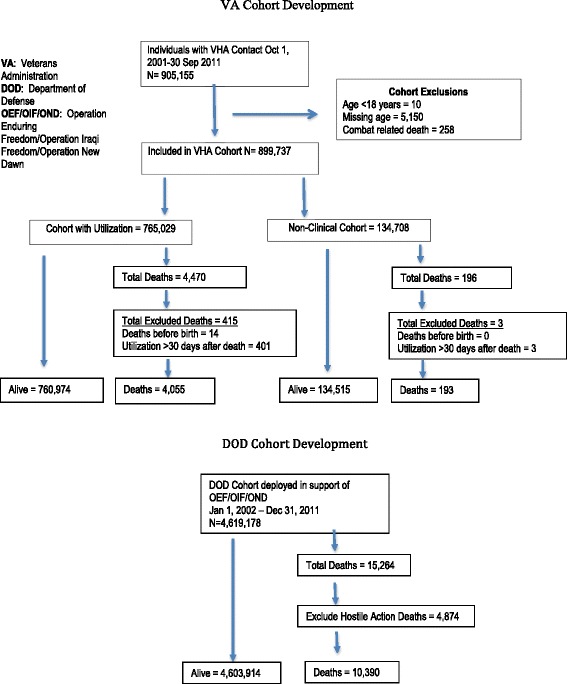
**Cohort development.**

We first identified individuals from the Roster file who had contact with the VA health care system at least once between October 1, 2001 and September 30, 2011 (n = 905,155). Veterans were excluded if they: 1) were <18 years of age by the end of Fiscal Year (FY)11, 2) had missing age information, or 3) had deaths determined to be combat-related. Our final cohort consisted of 899,737 individuals (see Figure 1). We then divided the cohort into groups based on VA utilization -- those who utilized VA versus those who had not by the end of FY11, to remove the confounding effect of clinical care-seeking on mortality differences, since veterans who use the VA are known to be less healthy than those who do not. After describing the full cohort and examining crude mortality rates, we removed the Coast Guard from comparisons because their extremely small numbers made estimates unreliable and unstable.

Our active duty military cohort was obtained from DRS and consisted of individuals deployed in support of OEF/OIF/OND from January 2002 through December 2011 (4,614,304 service members). As with VA data, we excluded persons whose deaths were determined to be combat-related.

### Outcomes

Mortality: For the VA cohort, we identified date of death using the VA Mini Vital Status file. In some cases, multiple and conflicting VA user records create misclassifications in mortality ascertainment [19]. We identified probable death misallocations and reclassified veterans identified as dead in the Vital Status File to alive if (1) their date of death occurred before their date of birth (N = 14) or (2) they had health service utilization more than 30 days after reported death (N = 402). Since VA enrollees were sometimes re-deployed after initial VA contact, we excluded persons whose date of death equaled or was less than the last day of their last military enlistment, since these deaths were likely due to combat. With these exclusions, deaths totaled 4,248 for the VA cohort. The total number of DOD non-combat deaths between January 2002 and December 2011 was 10,390 (see Figure 1).

Mortality rates for the US were calculated using 2002-2010 US population (N = 1,853,922,017) and deaths (N = 7,890,897) obtained from the CDC Wonder system [20]. US mortality rates were derived by sex, age, and race/ethnicity for those aged 18-72.

### Definition of other demographic characteristics

Demographic data obtained from the VA’s OEF/OIF/OND Roster file included: age, sex, race/ethnicity, education, rank, marital status, military service branch, military component, and year of military discharge. Similar demographic data on the DOD population were obtained from the DRS. Age is reported as the member’s age in 2010 and has been classified into categories using the PROC RANK procedure in SAS 9.2 to determine appropriate cut points. Ethnicity is reported separately from race in DOD data so comparisons can only be made by ethnicity or by race but not both.

### Analysis

We first described demographic and service-related factors within each of our cohorts using descriptive statistics. We then evaluated unadjusted associations between survivors and those who died using Chi-square tests. Finally, factors associated with mortality were examined by calculating the standardized mortality ratio (SMR) to control for the different age structures of the DOD, VA, and US populations. SMR calculations used indirect standardization [21] because of unstable mortality data (i.e., small numbers) in some segments of our study population. We calculated the SMR in two ways: (1) we applied mortality rates standardized to US age-, race-, and sex-specific mortality to the age structure of the VA and DOD populations to get an expected number of deaths; and (2) we applied mortality rates standardized to DOD age-, rank-, component-, and branch-specific mortality to the VA cohorts’ age, rank, component, and branch structure to get an expected number of deaths to identify whether mortality differences between VA and DOD cohorts were due to military-specific characteristics.

The ratio of actual to expected number of deaths estimated the SMR. An SMR greater than 1 indicates greater than expected mortality while an SMR less than 1 indicates lower than expected mortality. Significance was calculated using the standard error of the SMR where the number of observed deaths was over 100, written as: √O÷E. Where the observed number of deaths was less than 100, the upper and lower limits of the 95% confidence interval were calculated from the Poisson distribution as: (Poisson distribution lower limit)/E and (Poisson distribution upper limit)/E. Because we had limited information on the DOD cohort, we used population at risk rather than person-time at risk in our SMR calculations.

Additionally, because we used indirect standardization to compute the SMR, we cannot directly compare VA and DOD [22]. Results can only be compared to the US standard population (approximately 93% civilian) [5,23]. However, we did compute a directly standardized relative risk (DSRR) [23] using the SMR and the population standard for each age group to facilitate comparisons between groups, and we also compared VA cohorts using a DOD population standard on military-specific characteristics for which US data are unavailable.

## Results

Demographic and service characteristics of our VA cohorts, as well as our active duty military cohort with service between 2002-2011, are shown in Table 1. The DOD population is younger on average (mean age 27.2) compared to the VA cohorts (mean age 34.2-all VA cohort, 34.4-VA utilizers, and 33.1-VA non-utilizers). Relative to DOD, VA veterans were more likely to be male, married, and have some college education. VA data had much more missing race/ethnicity data compared to DOD. Because DOD race data include all ethnicities, it is difficult to compare DOD and VA except for Hispanic ethnicity. VA data indicate that Hispanics are more prevalent in the all-VA and VA-utilizer cohorts relative to DOD, while the VA non-utilizers have a slightly smaller proportion of Hispanics relative to DOD. Other notable findings include National Guard and Army members being over-represented in the VA cohorts relative to DOD, while Air Force and Navy personnel were under-represented.

**Table 1:** **Demographic characteristics: comparison of VA Total Population, VA Utilizers, and DOD OEF/OIF/OND populations, 2002–2011**

**Variable**	**Variable Veterans Administration (VA), 2002-2011**						**Department of Defense (DOD)**	
	**VA total population**		**VA utilizers**		**VA non-utilizers**			
	**N**	**%/mean (s.d.)**	**N**	**%/mean (s.d.)**	**N**	**%/mean (s.d.)**	**N**	**%/mean (s.d.)**
**All**	899,737		765,029		134,708		4,614,304	
**Age**								
<24	499,332	55.5%	421,226	55.0%	78,106	58.0%	2,735,575	59.3%
25-29	100,839	11.2%	85,866	11.2%	14,973	11.1%	602,127	13.0%
30-39	198,579	22.1%	169,845	22.2%	28,734	21.3%	841,902	18.2%
40-72	100,987	11.2%	88,092	11.5%	12,895	9.6%	432,643	9.4%
Missing	-	-	-	-	-	-	2,057	0.0%
Median Age 2002-2010		31.0		31.0		30.0		23.8
Mean Age 2002-2010		34.2 (9.6)		34.4 (9.5)		33.1 (9.7)		27.2 (10.7)
**Sex**								
Female	106,300	11.8%	92,919	12.1%	13,381	9.9%	775,155	16.8%
Male	793,437	88.2%	672,110	87.9%	121,327	90.1%	3,838,932	83.2%
Missing	-	-	-	-	-	-	217	0.0%
**Race** ^**1**^								
Hispanic	96,100	10.7%	84,830	11.1%	11,270	8.4%	397,942	8.6%
White, Non-Hispanic	546,952	60.8%	465,745	60.9%	81,207	60.3%	3,436,481	74.5%
Black, Non-Hispanic	122,505	13.6%	109,513	14.3%	12,992	9.6%	781,547	16.9%
Other, Non-Hispanic	35,800	4.0%	30,660	4.0%	5,140	3.8%	189,934	4.1%
Unknown	98,380	10.9%	74,281	9.7%	24,099	17.9%	206,342	4.5%
**Marital Status**								
Married	401,489	44.6%	343,400	44.9%	58,089	43.1%	1,511,015	32.7%
Not Married	498,095	55.4%	421,571	55.1%	76,524	56.9%	3,092,140	67.0%
Missing	153	0.0%	58	0.0%	95	0.0%	11,149	0.2%
**Education**								
< High School	13,862	1.50%	10,594	1.4%	3,268	2.4%	63,987	1.4%
High School	664,827	73.90%	572,804	74.9%	92,023	68.3%	3,530,639	76.5%
> High School	209,203	23.30%	171,737	22.4%	37,466	27.8%	879,414	19.1%
Missing	11,845	1.30%	9,894	1.3%	1,951	1.5%	140,264	3.0%
**Rank**								
Enlisted	811,905	90.2%	695,385	90.9%	116,520	86.5%	4,152,416	90.0%
Officer	78,185	8.7%	61,532	8.0%	16,653	12.4%	435,614	9.4%
Warrant Officer	9,647	1.1%	8,112	1.1%	1,535	1.1%	26,274	0.6%
**Component of Service**								
Active Duty	478,304	53.2%	434,618	56.8%	43,686	32.4%	2,925,780	63.4%
National Guard	260,006	28.9%	205,024	26.8%	54,982	40.8%	910,902	19.7%
Reserve	161,427	17.9%	125,387	16.4%	36,040	26.8%	777,622	16.9%
**Branch of Service**								
Army	553,267	61.5%	466,881	61.0%	86,386	64.1%	2,257,184	48.9%
Coast Guard	1,103	10.0%	821	0.1%	282	20.0%		0.0%
Air Force	112,573	12.5%	95,608	12.5%	16,965	12.6%	932,868	20.2%
Marines	116,393	12.9%	102,089	13.3%	14,304	10.6%	560,960	12.2%
Navy	116,401	12.9%	99,630	13.0%	16,771	12.5%	863,292	18.7%

^1^For DOD, race includes all ethnicities.

Table 2 shows the unadjusted mortality rates by demographic and military service characteristics in each of our cohorts. The average age at death was higher in the VA non-clinical population compared to active-duty military members (35.6 vs. 28.0). Chi-square tests indicated significant differences in mortality by age within each cohort, with the count of deaths highest for those 24 and younger and lowest for those 25-29 years of age among VA cohorts, and 24 and younger and 40+ for DOD members. Crude mortality rates increased with age for all groups and were higher among men compared to women in all cohorts. Mortality differed significantly by race/ethnicity, education, rank, service component, and branch of service for VA overall, VA utilizers, and DOD, but only rank and service component were significant for the VA non-utilizers. Finally, for all variables, the VA total population and VA utilizers had the highest crude rates. Within DOD, the unadjusted mortality rates were for persons with less than high school education while all VA cohorts had the highest crude rates for the oldest group in the age category, highlighting the importance of standardization.

**Table 2:** **VA and DOD unadjusted mortality rates by demographic and military service characteristics in OEF/OIF/OND Veterans, 2002-2011**

**Variable**	**VA Total population (N=899,737)**		**p-value**	**Crude mortality rate per 1,000**	**VA Utilizers (N=765,029)**		**p-value**	**Crude mortality rate per 1,000**	**VA Non-Utilizers (N=134,708)**		**p-value**	**Crude mortality rate per 1,000**	**Department of Defense (DOD) (N=4,614,304)**		**p-value**	**Crude mortality rate per 1,000**
	**Alive**	**Deceased**			**Alive**	**Deceased**			**Alive**	**Deceased**			**Alive**	**Deceased**		
**All**	895,489	4,248		4.72	760,974	4055		5.30	134,515	193		1.43	4,603,914	10,390		2.26
**Age**			<0.0001				<0.0001				<0.001				<0.0001	
<24	497,304	2,028		4.06	419,295	1,931		4.58	78,009	97		1.24	2,729,717	5,858		2.14
25-29	100,402	437		4.33	85,444	422		4.91	14,958	15		1.00	600,746	1,381		2.29
30-39	197,667	912		4.59	168,980	865		5.09	28,687	47		1.64	839,907	1,995		2.37
40-72	100,116	871		8.62	87,255	837		9.50	12,861	34		2.64	431,492	1,151		2.66
Missing	-	-			-	-		-	-	-		-	2,052	5		2.43
Mean Age 2011	34.2 (9.6)	37.2 (11.3)	<0.0001		34.4 (9.5)	37.2 (11.2)	<0.0001	-	33.1 (9.7)	35.6 (12.0)	<0.0001	-	27.2	28.0		-
**Sex**			<0.0001				<0.0001				<0.01				<0.0001	
Female	106,073	227		2.14	92,700	219		2.36	13,373	8		0.60	776,073	790		1.02
Male	789,416	4,021		5.07	668,274	3,836		5.71	121,142	185		1.52	3,827,798	9,600		2.50
Missing	-	-			-	-			-	-			43	-		-
**Race** ^**1**^			<0.0001				<0.0001				0.73				<0.0001	
Hispanic	95,779	321		3.34	84,522	308		3.63	11,257	13		1.15	397,157	785		1.97
White, Non-Hispanic	544,167	2,795		5.11	463,072	2,673		5.74	81,095	112		1.38	3,428,740	7,741		2.25
Black, Non-Hispanic	121,958	547		4.47	108,985	528		4.82	12,973	19		1.46	779,664	1,883		2.41
Other, Non-Hispanic	35,661	139		3.88	30,529	131		4.27	5,132	8		1.56	189,618	316		1.66
Unknown	97,924	456		4.64	73,866	415		5.59	24,058	41		1.70	205,892	450		2.18
**Marital Status**			0.41				0.54				0.66				<0.0001	
Married	399,569	1,920		4.78	341,560	1,840		5.36	58,009	80		1.38	1,507,356	3,659		2.42
Not Married	495,767	2,328		4.67	419,356	2,215		5.25	76,411	113		1.48	3,085,428	6,712		2.17
Missing	153	-		-	58	-		-	95	-		-	11,130	19		1.70
**Education**			<0.0001				<0.0001				0.09				<0.0001	
< High School	13,798	64		4.62	10,535	59		5.57	3,263	5		1.53	34,401	102		2.96
High School	661,503	3,324		5.00	569,628	3,176		5.54	91,875	148		1.61	3,214,570	8,243		2.56
> High School	208,402	801		3.83	170,976	761		4.43	37,426	40		1.07	1,271,707	1,773		1.39
Missing	11,786	59		4.98	9,835	59		5.96	1,951	-		-	83,236	272		3.26
**Rank**			<0.0001				<0.0001				0.01				<0.0001	
Enlisted	807,933	3,972		4.89	691,594	3,791		5.45	116,339	181		1.55	4,041,920	9,397		2.32
Officer	77,946	239		3.06	61,304	228		3.71	16,642	11		0.66	516,754	890		1.72
Warrant Officer	9,610	37		3.84	8,076	36		4.44	1,534	1		0.65	45,240	103		2.27
**Component of Service**			<0.01				<0.01				0.02				<0.0001	
Active Duty	475,942	2,362		4.94	432,328	2,290		5.27	43,614	72		1.65	2,917,552	8,228		2.81
Reserve	160,752	675		4.18	124,747	640		5.10	36,005	35		0.97	776,676	946		1.22
National Guard	258,795	1,211		4.66	203,899	1,125		5.49	54,896	86		1.56	909,686	1,216		1.33
**Branch of Service**			<0.0001				<0.01				0.93				<0.0001	
Army	550,546	2,721		4.92	464,287	2,594		5.56	86,259	127		1.47	2,252,081	5,103		2.26
Air Force	112,082	491		4.36	95,141	467		4.88	16,941	24		1.41	931,130	1,738		1.86
Marines	115,876	517		4.44	101,593	496		4.86	14,283	21		1.47	559,467	1,493		2.66
Coast Guard	1,103	3		2.71	-	-		-	-	-		-	-	-		-
Navy	115,885	516		4.43	99,135	495		4.97	16,750	21		1.25	861,236	2,056		2.38

^1^For DOD, race includes all ethnicities.

In Table 3 comparisons within cohorts can be made using the SMR while the directly standardized relative risk (DSRR) allows comparisons across groups. Overall, we found more deaths among VA veterans overall, VA utilizers, and DOD and fewer deaths among VA non-utilizers than expected. DOD mortality was 50% higher than the US standard, while all VA mortality was nearly three times higher, and the mortality of VA utilizers was more than three times higher. In contrast, mortality for VA non-utilizers did not differ significantly from the US population. Despite the overabundance of men in these cohorts, the SMR for men and women was similar for each cohort. We also saw a strongly negative association of mortality with age; increasing age is associated with lower mortality relative to the US population. For race and ethnicity, black non-Hispanic veterans had the lowest mortality for all VA cohorts, while black service members had the highest mortality in the DOD cohort (SMR 2.3). Across all characteristics, the all VA cohort consistently had DSRR values two to three times higher than DOD and VA non-utilizers. In contrast, VA non-utilizers had DSSR values approximately 30% lower than DOD.

**Table 3:** **Excess mortality in OEF/OIF/OND VA and DOD veterans compared to the US population, 2002–2011**

**Variable**	**VA Total Population (N=899,737)**			**VA Utilizers (N=765,029)**			**VA Non-Utilizers (N=134,708)**			**Department of Defense (DOD) (N=4,614,304)**		
	**SMR**	**95% Confidence interval**	**DSRR**	**SMR**	**95% Confidence interval**	**DSRR**	**SMR**	**95% Confidence interval**	**DSRR**	**SMR**	**95% Confidence interval**	**DSRR**
**All**	2.84	2.75-2.92	2.56	3.15	3.1-3.25	2.86	0.92	0.79-1.05	0.79	1.47	1.44-1.49	1.19
**Age**												
<24	4.69	4.48-4.89	-	5.29	5.05-5.53	-	1.43	1.16-1.75	-	2.47	2.41-2.53	-
25-29	4.38	4.38	-	4.96	4.49-5.44	-	1.01	0.57-1.67	-	2.32	2.19-2.44	-
30-39	3.49	3.26-3.71	-	3.87	3.61-4.13	-	1.24	0.91-1.65	-	1.80	1.72-1.88	-
40-72	1.24	1.16-1.32	-	1.37	1.27-1.46	-	0.38	0.26-0.53	-	0.38	0.36-0.40	-
**Sex**												
Female	2.15	1.87-2.43	2.05	2.36	2.05-2.67	2.24	0.64	0.28-1.26	0.66	1.10	1.02-1.18	0.94
Male	2.28	2.21-2.35	2.04	2.54	2.46-2.62	2.28	0.73	0.62-0.83	0.62	1.20	1.18-1.23	0.98
**Race** ^**1**^												
Hispanic	2.92	2.60-3.24	2.81	3.13	2.78-3.48	3.00	1.14	0.61-1.95	1.17	1.87	1.74-2.00	1.62
White, Non-Hispanic	3.36	3.23-3.48	2.77	3.75	3.61-3.89	3.11	0.96	0.78-1.14	0.75	1.09	1.07-1.12	0.82
Black, Non-Hispanic	1.55	1.42-1.68	1.60	1.64	1.50-1.78	1.72	0.62	0.37-0.97	0.57	2.33	2.22-2.43	2.39
Other, Non-Hispanic	4.01	3.34-4.67	3.98	4.31	3.58-5.05	4.40	1.85	0.80-3.65	1.23	1.56	1.39-1.73	0.95
Unknown	2.29	2.08-2.50	2.34	2.71	2.45-2.97	2.83	0.88	0.64-1.20	0.88	1.35	1.23-1.47	1.10

Indirectly Standardized Mortality Ratios (SMR) and Directly Standardized Relative Risks (DSRR).

^1^For DOD, race includes all ethnicities.

Finally, we examined excess mortality in VA cohorts compared with DOD (Table 4). Consistent with prior analyses, we found that the overall mortality was two times higher in the VA-all and VA-utilizer population and nearly 40% lower in VA non-utilizers than in active duty personnel. We also observe an SMR twice the DOD standard for enlisted personnel in the VA-all and VA-utilizers but 30% lower for the VA non-utilizers. For Officers and Warrant Officers, this effect size is diminished relative to DOD. The SMRs are still elevated for Officers relative to the DOD standard, but the differences are insignificant for Warrant Officers in both the VA-all and VA utilizer populations. In contrast, VA non-utilizers’ SMR values also strengthen for Officers and Warrant Officers but in the opposite direction. Mortality in this cohort is 70% lower for Officers and 86% lower for Warrant Officers relative to DOD.

**Table 4:** **Excess mortality in OEF/OIF/OND VA veterans compared to the DOD population**

**Variable**	**VA Total Population (N=899,737)**			**VA Utilizers, (N=765,029)**			**VA Non-Utilizers, (N=134,708)**		
	**SMR**	**95% Confidence interval**	**DSRR**	**SMR**	**95% Confidence interval**	**DSRR**	**SMR**	**95% Confidence interval**	**DSRR**
**All**	2.08	2.02-2.15	2.03	2.34	2.26-2.41	2.28	0.63	0.55-0.72	0.62
**Rank**									
Enlisted	2.15	2.08-2.21	2.08	2.39	2.31-2.46	2.31	0.69	0.59-0.79	0.67
Officer	1.51	1.32-1.70	1.40	1.82	1.58-2.05	1.68	0.33	0.17-0.60	0.33
Warrant Officer	0.90	0.63-1.23	1.04	1.05	0.74-1.46	1.17	0.14	0.00-0.78	0.26
**Component of Service**									
Active	1.75	1.68-1.82	1.75	1.87	1.79-1.94	1.87	0.59	0.52-0.82	0.59
Guard	3.42	3.23-3.61	3.38	3.95	3.72-4.18	3.94	1.24	0.99-1.53	1.28
Reserve	3.36	3.11-3.61	3.46	4.04	3.73-4.36	4.24	0.82	0.57-1.13	0.8
**Branch of Service**									
Air Force	2.32	2.11-2.52	2.11	2.60	2.36-2.83	2.38	0.75	0.48-1.12	0.58
Army	2.15	2.07-2.23	2.10	2.43	2.33-2.52	2.37	0.65	0.54-0.77	0.65
Marines	1.67	1.53-1.82	1.68	1.83	1.67-1.99	1.84	0.55	0.34-0.85	0.56
Navy	1.83	1.67-1.99	1.75	2.05	1.87-2.23	1.97	0.52	0.32-0.79	0.47

Indirectly Standardized Mortality Ratios (SMR), 2002-2011.

Both Guard and Reserve have SMR values three to four times higher than the DOD standard for the VA-all and VA-utilizers, but VA non-utilizers have non-significant differences in SMR values. Army and Air Force personnel in the VA-all and VA-utilizer cohorts had SMR values twice the DOD standard, with SMRs for the Marines and Navy approaching that value in the VA-all and for Marines only in the VA-utilizer cohort. The SMR value for Navy personnel was twice that of the DOD standard in the VA-utilizer cohort. In contrast, the VA non-utilizer cohort had insignificant SMR values for Air Force and SMR values 48% to 35% lower than the DOD standard for the other services. Similar to SMR values, DSRR values are largest in VA-utilizers and lowest in VA non-utilizers. There really were no substantial differences between SMR values and DSRR values, and the changes that did arise in DSRR to compare across cohorts did not change the direction of any of the relationships.

## Discussion

While several studies have linked military service to a HSE, [9,13,24-26] to our knowledge, none of them have assessed the HSE in US OEF/OIF/OND veterans. Our results find no evidence of HSE in veterans of Iraq and Afghanistan and suggest that this cohort of US veterans have either equivalent or higher than expected mortality compared to the general US population.

Veteran cohorts have generally had better survival rates than the population at large due primarily to higher fitness standards required for entry to the military and ready access to routine medical care. However, we find that there has been deterioration in the military mortality advantage for active duty members. This deterioration is less visible in those who enroll in the VA health care system but who had not sought care by 2011, in contrast to being more visible in the VA-utilizers. Projections by the VA show a greater reliance by OEF/OIF/OND on VA, with an increase of 36% in outpatient visits expected for this veteran cohort [27]. This projection is supported by our findings of much higher mortality among the VA-utilizer cohort than DOD, suggesting that selection of VA care, especially at younger ages, was associated with a higher illness burden than in the non-utilizing VA cohort.

Whereas the literature indicates that HWE and HSE should be strongest at the youngest ages, we found a negative mortality-age gradient. Mortality relative to the US population was higher in the youngest veterans and lowest in the oldest veterans. In the non-utilizing VA group, these differences were statistically significant only at the extremes of age where in VA-utilizers all ages were significantly different from the US standard. Since increased age is associated with increased length of military service, the lower mortality in the oldest ages suggests evidence of a Healthy Soldier Survivor Effect (HSSE) – increased time in military service may be providing beneficial health effects. Higher than expected mortality at the youngest ages parallels the association between shorter duration of employment and elevated mortality in HWE studies of the chemical industry [28]. Additionally, evidence suggests that combat experience may lead younger soldiers to engage in risky and dangerous behaviors such as speeding, drinking and driving, and failure to wear seat belts [7]. Therefore, the elevated mortality at the youngest ages may be attributable to risk-taking behaviors, which we know are higher in this OEF/OIF/OND cohort than earlier military cohorts [24,25], while lower mortality at older ages is associated with HSSE. We should also note that over 50% of veterans in our study ended their final deployment in FY2007. Thus, our follow-up time was both censored and varied. However, studies of Persian Gulf veterans with similar follow-up times found a HSE [3].

Sex is a known modifier of the HWE, yet we found only slight differences between men and women. Women had no combat role so women should have had significantly lower mortality than men consistent with the literature. However, the lack of difference may be due to the fact that without a front line on the battle field, anyone deployed to Iraq or Afghanistan was at risk of assault or attack, even those providing non-combat support [26,29].

Prior literature also showed the HWE was highest for non-whites [5]. Our results indicate the HSE is strongest for Non-Hispanic blacks but only in the VA non-utilizer cohort. In contrast, Non-Hispanic Other groups had the highest SMR in the VA-all and VA-utilizer cohorts, while within DOD, the black SMR was the highest among all races and shows two times the risk of mortality relative to the US black population. While blacks have a greater likelihood of being assigned to non-combat positions [30,31] this would do little to reduce active duty mortality when anyone deployed in theater would be at risk for assault [26,29]. However, long-term mortality might be reduced if veterans were not exposed to direct combat stress and the health conditions that stress creates.

The lack of an HSE in the DOD cohort might be due to the method of selection into the military. Military entrance requirements may also have played a role in our HSE outcomes, since entrance standards were relaxed to meet service recruitment goals for the Afghanistan and Iraq conflicts. Potential recruits who exceeded established weight standards, [32] scored lower on military aptitude examinations, had criminal and medical waivers, or lacked high school diplomas were allowed to enlist. [33-35] The lowering of education and testing standards is associated with difficulties in training and subsequent poor work performance [36], while lower education is consistently associated with higher mortality [37].

### Limitations

Several limitations are noted. First, the VA portion of this data represents only those OEF/OIF/OND veterans who have an existing relationship with VA. The VA enrollee population is not representative of the entire OEF/OIF/OND veteran population. Second, the potential for counting deaths twice – once for the VA and once for DOD – does exist, but we do not think this is a major issue as (1) active duty soldiers, who represent the majority of those who served in OEF/OIF/OND, would only transition to the VA if they were discharged from DOD alive; (2) Guard/Reserve forces were more likely to have been discharged from active duty and then recalled to active duty, which we attempted to control for by removing combat-related deaths from both the numerator and denominator in these analyses.

Third, we know very little about those who receive care outside of the VA. Their mortality experience may be very different. Fourth, our measure of death only reflects all-cause mortality. Future research will explicate the cause of death and examine the predictors of mortality in depth. This will be very important as we expand our follow-up period, since with new medical technologies designed to increase survival and decrease mortality in wounded veterans, the implications for mortality in the long-term may be quite different than in previous military cohorts. Fifth, these data were cross-sectional in nature and there was some variability in follow-up time. Future research will control for the period of time in VA care. Sixth, we recognize that a Healthy Warrior Effect (only healthy soldiers are deployed to combat) may be obscuring some mortality that we are attributing to OEF/OIF/OND deployment. We hope to obtain VA data that will allow us to determine who served in combat zones and who did not as well as the number of deployments for each subject so that we may control for different or repeated exposure. Seventh, we also recognize that using the population at risk rather than time at risk doesn’t allow us to control for varying lengths of time at risk. Again, we do not have these data from DOD and only very broadly from VA. To date, these data have only been available through survey research to us. We hope to identify administrative data resources for this information from both DOD and VA so that we can control for varying follow-up length. Eighth, mortality follow-up differed for VA (through October 2011) and DOD (through December 2011), underestimating observed mortality gaps. Finally, we were limited in our use of the DRS data, so some comparisons between VA and DOD (i.e., race and ethnicity) were not possible. Despite these limitations, the results presented here elucidate the HSE in a previously unstudied cohort of veterans.

## Conclusion

In summary, no HSE was evident in these cohorts of Iraq and Afghanistan veterans for all-cause mortality. Overall, the consistent and persistent military mortality advantage has eroded in VA cohorts although is still evident by sex, oldest age, and in some categories of race/ethnicity, but only in the VA non-utilizing cohort. The HSE has been eliminated overall in DOD, VA-all, and VA-utilizers but still appears at the oldest ages (HSSE) for DOD and VA non-utilizers. This HSE reversal may be due to repeated and prolonged deployments, a strong reliance on Guard and Reserve forces, and/or survival from injuries that would have meant death in earlier conflicts. This research highlights evidence that the OEF/OIF/OND military mortality experience is more complex than first thought. A modeling approach adjusting for covariates such as time in service, SES, and combat or in-theater exposure would provide insight into our results. Finally, examining specific causes of death would yield clues to our finding of an eroding military mortality advantage, which is important to military workforce and VA planning.

## Abbreviations

DMDC: Defense Manpower Data Center
DOD: Department of Defense
DRS: Defense Manpower Data Center Report System
DSRR: Directly Standardized Relative Risk
HSE: Healthy Soldier Effect
HSSE: Healthy Soldier Survivor Effect
HWE: Healthy Worker Effect
OEF/OIF/OND: Operation Enduring Freedom/Operation Iraqi Freedom/Operation New Dawn
SMR: Standardized Mortality Ratio
US: United States
VA: Veterans Administration
